# The chlamydial periplasmic stress response serine protease cHtrA is secreted into host cell cytosol

**DOI:** 10.1186/1471-2180-11-87

**Published:** 2011-04-28

**Authors:** Xiang Wu, Lei Lei, Siqi Gong, Ding Chen, Rhonda Flores, Guangming Zhong

**Affiliations:** 1 Department of Microbiology and Immunology, University of Texas Health Science Center at San Antonio, 7703 Floyd Curl Drive, San Antonio, TX 78229, USA

**Keywords:** *Chlamydia trachomatis*, cHtrA, serine protease, secreted protein

## Abstract

**Background:**

The periplasmic High Temperature Requirement protein A (HtrA) plays important roles in bacterial protein folding and stress responses. However, the role of chlamydial HtrA (cHtrA) in chlamydial pathogenesis is not clear.

**Results:**

The cHtrA was detected both inside and outside the chlamydial inclusions. The detection was specific since both polyclonal and monoclonal anti-cHtrA antibodies revealed similar intracellular labeling patterns that were only removed by absorption with cHtrA but not control fusion proteins. In a Western blot assay, the anti-cHtrA antibodies detected the endogenous cHtrA in Chlamydia-infected cells without cross-reacting with any other chlamydial or host cell antigens. Fractionation of the infected cells revealed cHtrA in the host cell cytosol fraction. The periplasmic cHtrA protein appeared to be actively secreted into host cell cytosol since no other chlamydial periplasmic proteins were detected in the host cell cytoplasm. Most chlamydial species secreted cHtrA into host cell cytosol and the secretion was not inhibitable by a type III secretion inhibitor.

**Conclusion:**

Since it is hypothesized that chlamydial organisms possess a proteolysis strategy to manipulate host cell signaling pathways, secretion of the serine protease cHtrA into host cell cytosol suggests that the periplasmic cHtrA may also play an important role in chlamydial interactions with host cells.

## Background

The genus *Chlamydia *consists of multiple obligate intracellular bacterial species that infect both humans and animals. The *C. trachomatis *organisms infect human ocular (serovars A to C) and urogenital/colorectal (serovars D to K & L1 to L3) epithelial tissues, causing trachoma [1] and sexually transmitted diseases [2-4] respectively; The *C. pneumoniae *organisms invade human respiratory system, not only causing respiratory diseases but also exacerbating pathologies in cardiovascular system [5-7]; *C. muridarum *(formerly known as *C. trachomatis *mouse pneumonitis agent, designated as MoPn; ref: [8]), although causing no known diseases in humans, has been used as a model pathogen for studying chlamydial pathogenesis and immune responses; The *C. psittaci *6BC organisms that naturally infect birds can cause severe pneumonia in humans [9] while the *C. caviae *GPIC organisms can infect ocular and urogenital tissues in guinea pig [10]. Despite the differences in host range, tissue tropism, disease processes, all chlamydial species share similar genome sequences [8,10,11] and possess a common intracellular growth cycle with distinct biphasic stages [12]. A chlamydial infection starts with the invasion of an epithelial cell by an infectious elementary body (EB). The internalized EB rapidly develops into a noninfectious but metabolically active reticulate body (RB) that undergoes multiplication. The progeny RBs then differentiate back into EBs for spreading to new cells. All chlamydial biosynthesis activities are restricted within a cytoplasmic vacuole known as inclusion [12].

During the intravacoular developmental cycle, chlamydial organisms have to take up nutrients and energy from host cells [13-16] and maintain the integrity of the host cells [17]. To achieve these goals, chlamydial organisms have evolved the ability to secrete proteins into the inclusion membrane [18,19] and host cell cytoplasm [17,20,21]. Identifying the chlamydial secretion proteins has greatly facilitated the understanding of chlamydial pathogenic mechanisms [20,22-31]. CPAF, a chlamydial protease/proteasome-like activity factor that is now known as a serine protease [32,33], was found to secrete into host cell cytosol more than a decade ago [26]. CPAF can degrade a wide array of host proteins including cytokeratins for facilitating chlamydial inclusion expansion [34-36], transcriptional factors required for MHC antigen expression for evading immune detection [37,38] and BH3-only domain proteins for blocking apoptosis [39,40]. Another example of chlamydia-secreted proteins is the chlamydial tail-specific protease that has been found to dampen the inflammatory responses by cleaving host NF-κB molecules [41,42]. These observations have led to the hypothesis that *Chlamydia *may have evolved a proteolysis strategy for manipulating host cell signaling pathways [17].

Among the several dozens of putative proteases encoded by chlamydial genomes [11,43], the chlamydial HtrA (cHtrA) is a most conserved protease. HtrA from eukaryotic and prokaryotic species exhibits both chaperone and proteolytic activities [44,45] with a broad proteolytic substrate specificity [44,45]. HtrA is a hexamer formed by staggered association of trimeric rings and access to the proteolytic sites in central cavity is controlled by 12 PDZ domains in the sidewall [46,47]. In eukaryotic cells, HtrA responds to unfolded proteins in the endoplasmic reticulum (ER) by cleaving and releasing the ER membrane-anchored transcription factors ATF6 and SREBP into nucleus to activate the expression of proteins required for the unfolded protein response and cholesterol biosynthesis [48,49]. In bacteria, the periplasmic HtrA, in response to the binding of C-terminal peptides from unfolded/reduced outer membrane proteins, cleaves and releases the σ^E^-factor to activate stress response genes [50]. Since HtrA is required for bacterial survival under high temperature, it is called High Temperature Requirement (Htr) protein [51]. Although both the tertiary structure and the function of HtrA are well known, the role of cHtrA in chlamydial pathogenesis remains unclear. In the current study, we have localized cHtrA both in the chlamydial inclusions and the host cell cytosol. The specificity of the antibody labeling and cytosolic localization of cHtrA were confirmed in independent assays. The secretion of the periplasmic cHtrA into host cell cytosol appeared to be an active/selective process since no other chlamydial periplasmic proteins were detected outside the chlamydial inclusions. Thus, the chlamydial periplasmic cHtrA may also contribute to the chlamydial proteolysis strategies for manipulating host cell signaling pathways.

## Methods

### 1. Chlamydial infection

The following chlamydial organisms were used in the current study: *C. trachomatis *serovars A/HAR-13, B/HAR-36, Ba/Ap-2, C/UW-1, D/UW-3/Cx, E/UW-5/CX), F/IC-Cal-3, H/UW-43/Cx, I/UW-12/Ur, K/UW-31/Cx, L1/LGV-440, L2/LGV-434/Bu & L3/LGV-404, *C. muridarum *(Nigg), *C. pneumoniae *(AR39), *C. caviae *(GPIC) &*C. psittaci *(6BC). All chlamydial organisms were either purchased from ATCC (Manassas, VA) or acquired from Dr. Harlan Caldwell at the Rocky Mountain Laboratory, NIAID/NIH (Hamilton, MT) or Dr. Ted Kou at the University of Washington (Seattle, WA). The chlamydial organisms were propagated, purified, aliquoted and stored as described previously [26]. All chlamydial organisms were routinely checked for mycoplasma contamination. For infection, HeLa cells (human cervical carcinoma epithelial cells, ATCC cat# CCL2) grown in either 24 well plates with coverslips or tissue flasks containing DMEM (GIBCO BRL, Rockville, MD) with 10% fetal calf serum (FCS; GIBCO BRL) at 37°C in an incubator supplied with 5% CO2 were inoculated with chlamydial organisms. The infected cultures were processed at various time points after infection for either immunofluorescence assays or Western blot analysis as described below. In some experiments, at 6 hours after infection, the cultures were treated with a C1 compound [N'-(3,5-dibromo-2-hydroxybenzylidene)-4-nitrobenzohydrazide, cat#5113023, ChemBridge, San Diego, CA], a small molecule known to inhibit *Yersinia *type III secretion system (T3SS) and block chlamydial growth [52]. The treated cultures were processed for immunofluorescence microscopy analysis at 36 hours after infection. The C1 compound was dissolved in dimethyl sulfoxide (DMSO; Sigma, St Luis, MO) at a stock concentration of 50 mM and diluted into culture medium at a final concentration of 50 μM with 0.1% DMSO.

### 2. Chlamydial gene cloning, fusion protein expression and antibody production

The ORF CT823 (cHtrA) from *C. trachomatis *serovar D organisms was cloned into pGEX vectors (Amersham Pharmacia Biotech, Inc., Piscataway, NJ). The following primers were used for cloning the ORF: cHtrA forward primer, 5'-CGC-GGATCC (BamHI)-ATGATGAAAAGATTATTATGTGTG-3', cHtrA back primer, 5'-TTTTCCTTTT-GCGGCCGC(NotI)-CTACTCGTCTGATTTCAAGAC-3'. The ORF was expressed as a fusion protein with glutathione-S-transferase (GST) fused to the N-terminus as previously described [53]. Expression of the fusion protein was induced with isopropyl-beta-D-thiogalactoside (IPTG; Invitrogen, Carlsbad, CA) and the fusion proteins were extracted by lysing the bacteria via sonication in a Triton-X100 lysis buffer (1%TritonX-100, 1 mM PMSF, 75 units/ml of Aprotinin, 20 μM Leupeptin and 1.6 μM Pepstatin, all from Sigma). After a high-speed centrifugation to remove debris, the fusion protein was purified using glutathione-conjugated agarose beads (Pharmacia) and the purified protein was used to immunize mice for producing antibodies, including monoclonal antibodies (mAbs), as described previously [53-55]. The mouse antibodies against GST-CT067, GST-CT539 and GST-CT783 were produced similarly. The fusion protein-specific antibodies were used to localize endogenous proteins in *C. trachomatis*-infected cells via an indirect immunofluorescence assay and to detect endogenous proteins using a Western blot assay. All mouse anti-GST fusion protein antibodies were preabsorbed with bacterial lysates containing GST alone before any applications. In some experiments, the GST fusion proteins bound onto the glutathione-agarose beads were also used to absorb the mouse antibodies to confirm antibody specificities.

### 3. Immunofluorescence assay

The immunofluorescence assay was carried out as described previously [55]. Briefly, HeLa cells grown on coverslips were fixed with 2% paraformaldehyde (Sigma, St. Luis, MO) for 30 min at room temperature, followed by permeabilization with 2% saponin (Sigma) for an additional 30 min. After washing and blocking, the cell samples were subjected to antibody and chemical staining. Hoechst (blue, Sigma) was used to visualize DNA. A rabbit anti-chlamydial organism antibody (R1L2, raised with *C. trachomatis *L2 organisms, unpublished data) or anti-IncA from *C. trachomatis *[kindly provided by Ted Hackstadt. Laboratory of Intracellular Parasites, Rocky Mountain Laboratories, NIAID, NIH, Hamilton, Montana; [56]], *C. pneumoniae *or *C. psittaci *(both current study) plus a goat anti-rabbit IgG secondary antibody conjugated with Cy2 (green; Jackson ImmunoResearch Laboratories, Inc., West Grove, PA) was used to visualize chlamydial organisms or inclusion membrane. The various mouse antibodies plus a goat anti-mouse IgG conjugated with Cy3 (red; Jackson ImmunoResearch, West Grove, PA) were used to visualize the corresponding antigens. The mouse antibodies used included: polyclonal antibodies (pAbs) made against GST-CT823 (HtrA), GST-CT783, GST-CT621, GST-CT539, GST-CT067 (all current study) and mAbs 6A2 against HtrA (current study), 100a against CPAF [26], BB2 against IncA (CT119) & 1L11C3 against chlamydial HSP60 (unpublished data). All primary antibodies were preabsorbed with a bacterial lysate containing GST alone before use. In addition, for some experiments, the primary antibodies were absorbed with either the corresponding or heterologous fusion proteins immobilized onto glutathione-conjugated agarose beads (Pharmacia). The absorption was carried out by incubating the antibodies with bead-immobilized antigens for 1 h at room temperature (RT) or overnight at 4°C followed by pelleting the beads. The remaining supernatants were used for immunostaining. The immunofluorescence images were acquired using an Olympus AX-70 fluorescence microscope equipped with multiple filter sets and Simple PCI imaging software (Olympus, Melville, NY) as described previously [40]. An Olympus FluoView laser confocal microscope (Olympus, Center Valley, PA) was used to further analyze some of the immunofluorescence samples at the University of Texas Health Science Center at San Antonio institutional core facility as described previously [29]. The images were processed using Adobe Photoshop (Adobe Systems, San Jose, CA).

### 4. Western blot assay

The Western blot assay was carried out as described elsewhere [38,55]. Briefly, HeLa cells with or without *C. trachomatis *infection and with or without fractionation (into pellet and S100 fractions), purified chlamydial RB and EB organisms, GST fusion proteins or fractionated bacterial periplasmic or cytosolic samples were resolved in SDS polyacrylamide gels. The resolved protein bands were transferred to nitrocellulose membranes for antibody detection. The primary antibodies used included: mouse pAb and mAb 6A2 against cHtrA, mouse pAb against CT067 (all current study), mAb 100a against CPAF [26], mAb MC22 against chlamydial major outer membrane protein [MOMP; ref [26]], mAb W27 against host cell HSP70 (cat#Sc-24, Santa Cruz Biotechnology, CA), mAb against FLAG tag (cat#F3165, Sigma, St. Luis, MO) and rabbit polyclonal antibody against bacterial GroEL (cat#G6532, Sigma, St. Luis, MO). The anti-MOMP antibody was used to ensure that all lanes with chlamydial organism-containing samples were loaded with equivalent amounts of the organisms while the lanes without chlamydial organism samples should be negative for MOMP. The anti-HSP70 antibody was used to make sure that equal amounts of normal HeLa and *Chlamydia*-infected HeLa samples were loaded and to also check whether the cytosolic fractions are contaminated with components from the pellet fractions during cellular fractionation (see below). All primary antibodies used in the current study were pre-absorbed with an excess amount of bacterial lysates containing the GST alone. The primary antibody binding was probed with an HRP (horse radish peroxidase)-conjugated goat anti-mouse IgG secondary antibody (Jackson ImmunoResearch, West Grove, PA) and visualized with an enhanced chemiluminescence (ECL) kit (Santa Cruz Biotech). Some of the *C. trachomatis*-infected HeLa cell (Ct-HeLa) samples were fractionated into pellet (containing host cell nuclei and chlamydial inclusions) and cytosolic fraction (S100, containing *Chlamydia*-secreted proteins) as described previously [26,29]. Briefly, cell samples were collected by centrifugation at 600 *g *for 10 min at 4°C. The cell pellets were washed once with ice-cold PBS and resuspended with five volumes of buffer A (20 mM Hepes-KOH, pH 7.5, 10 mM KCl, 1.5 mM MgCl_2_, 1 mM sodium EDTA, 1 mM sodium EGTA, 1 mM dithiothreitol, and 0.1 mM phenylmethylsulfoyl fluoride) containing 250 mM sucrose on ice for 15 min. The cells were homogenized with 10 to 15 strokes using a number 22 kontes douncer with the B pestle (Kontes Glass Company, Vineland, NJ) to break cytoplasmic membrane but without breaking inclusion/nuclear membrane. The integrity of cytoplasmic and inclusion/nuclear membranes was monitored microscopically by smearing an aliquot of the homogenates on a slide. The final homogenates were centrifuged twice at 750 *g *for 10 min at 4°C to pellet inclusions/nuclei. The pellets from both centrifugations were combined and washed once with cold PBS and stored as pellet fraction. The supernatants were centrifuged at 10,000 *g *for 15 min at 4°C followed by a further centrifugation at 100,000 *g *for 1 h at 4°C. The resulting supernatants were designated as S100 or cytosolic fraction. The chlamydial organisms were purified as described previously [43]. The RB organisms were purified from 24 h cultures while the EB organisms from 40 to 50 h cultures. The bacterial cell fraction samples were prepared as the following: a pellet from 10 ml bacteria culture was washed with ice-cold PBS once and pelleted again by centrifugation at 3000 rmp × 10 min at 4°C. The pelleted bacterial cells were resuspended in 0.5 ml of a Periplasting buffer containing 20 mM Tris-HCl (pH7.5), 20% sucrose (cat#SX1075-1, EMD Chemicals Inc., Gibbstown, NJ), 1 mM EDTA (cat#E5134, Sigma), 3 mg/ml lysozyme (cat#100834, MP biomedicals, Solon, Ohio). After incubating on ice for 5 min, 0.5 ml ice-cold distilled water was added to the suspension and mixed by pipetting up and down. After incubating on ice for another 5 min, the mixture was pelleted by centrifugation at 12,000 g for 2 min at 4°C. The periplasmic fraction (per) in the supernatant was collected to a new tube while the cytoplasmic proteins (cyt) in the remaining pellet were resuspended in 1 ml Periplasting buffer. Both per & cyt fractions were used on the Western blot assay.

### 5. BCIP Assay

To construct the plasmid pFLAG-CTC-cHtrAss-'PhoA, a 69 bp DNA sequence coding for the HtrA signal peptide (M1-S23, designated as cHtrAss, with restriction enzyme sites of XhoI/BamHI) was amplified from *Chlamydia trachomatis *serovar D genome and 1400 bp DNA sequence coding for 'PhoA (BamHI/KpnI) was amplified from pFLAG-CTC-CPAFss-'PhoA plasmid, both 69 bp HtrA and 1400 bp 'PhoA were inserted into the XhoI/KpnI sites of the plasmid pFLAG-CTC (cat#E8408, sigma; 'PhoA stands for mature PhoA without the signal peptide). The DH5a bacterial strain (Invitrogen, Carlsbad, CA) was used to express the plasmids. The products from all the three plasmids (pFLAG-PhoA, pFLAG-'PhoA & pFLAG-HtrAss-'PhoA) contain a FLAG tag fused to the C-terminus of PhoA. For BCIP assay, bacterial cells were grown in LB supplemented with the corresponding selection antibiotics at 37°C overnight. The overnight cultures were streaked onto LB agar containing the same selection antibiotics and 50 μg/ml 5-bromo-4-chloro-3-indolyl phosphate (BCIP, cat# B6149, Sigma) and the plates were incubated at 30°C for 2 days. The bacterial colonies that are capable of exporting mature PhoA into periplasm turn blue while the colonies incapable of doing so remain white.

## Results

### 1. Chlamydial HtrA is localized in both chlamydial inclusion and host cell cytosol

**Figure 1:**
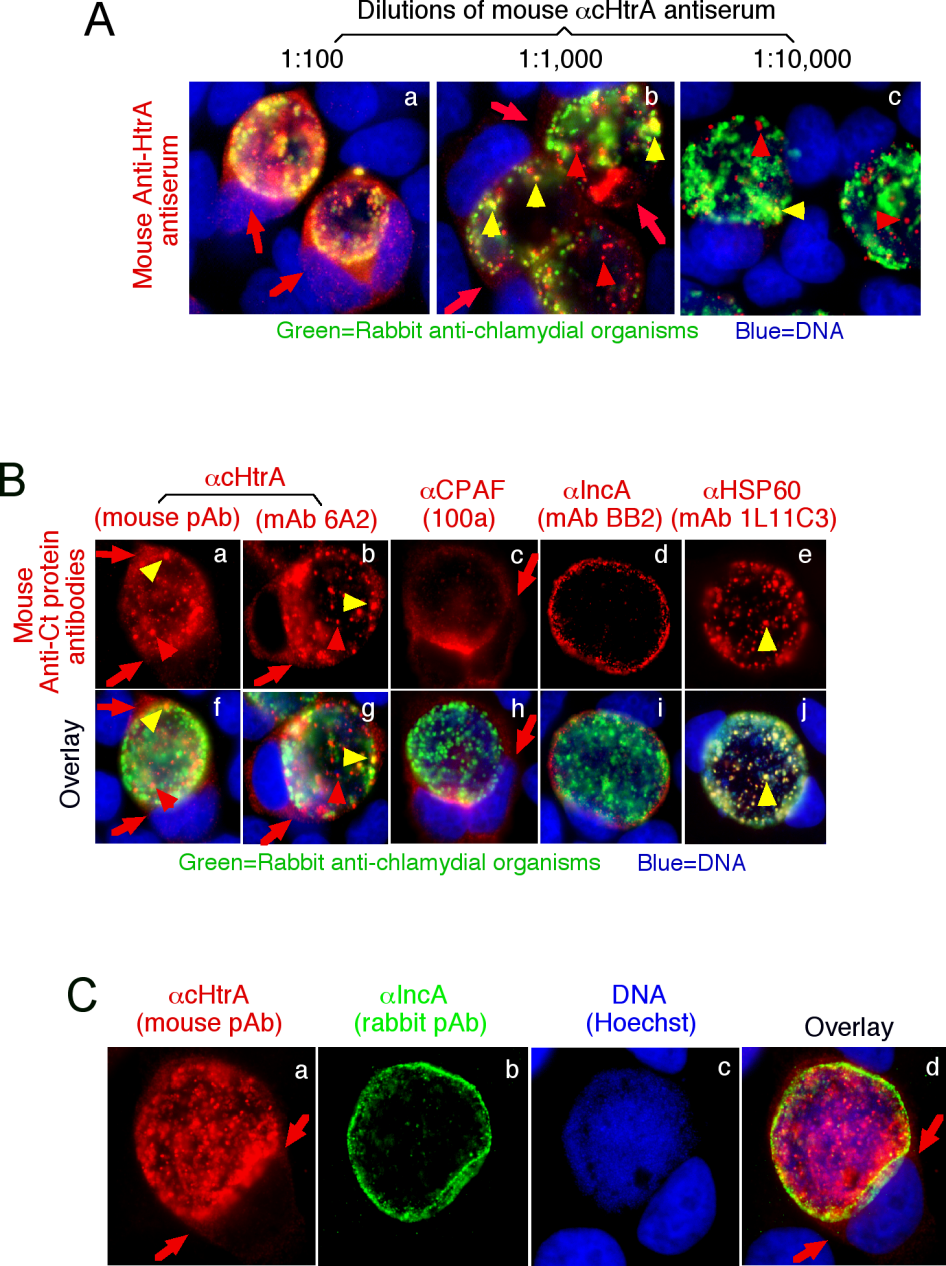
**Detection of cHtrA protease in the cytosol of *C. trachomatis*-infected cells**. HeLa cells infected with *C. trachomatis *L2 organisms were processed for co-staining with mouse antibodies visualized with a goat anti-mouse IgG conjugated with Cy3 (red), rabbit antibodies visualized with a Cy2-conjugated goat anti-rabbit IgG (green) and the DNA dye Hoechst (blue). The mouse antibodies included an anti-cHtrA (CT823) antiserum (raised with GST-cHtrA fusion protein) at various dilutions (**A**), the anti-cHtrA antiserum at 1:1000 dilution (**B**, panels a & f), mAb 6A2 (b & g, also raised with the GST-cHtrA fusion protein), mAb (100a) against CPAF (c & h), mAb (BB2) against IncA (d & i) and mAb (1L11C3) against HSP60 (e & j). The mouse anti-cHtrA staining (red) was also co-labeled with a rabbit anti-IncA antibody (green; **C**). Note that the anti-cHtrA antibodies detected signals both inside the chlamydial inclusions with (yellow arrowheads) or without (red arrowheads) overlapping with the chlamydial organisms and in the host cell cytosol (red arrows) while the anti-CPAF antibody mainly detected signals in the host cell cytosol.

A mouse antiserum raised with GST-cHtrA fusion protein detected the endogenous cHtrA protein both inside and outside of the chlamydial inclusions in *C. trachomatis*-infected HeLa cells (Figure 1A). The amount of intra-inclusion labeling appeared to be greater since the labeling in the host cell cytosol (outside inclusions) disappeared first as the dilution of the antiserum increased. Interestingly, some of the cHtrA-positive intra-inclusion granules appeared to be distinct from *C. trachomatis *organisms, suggesting that a portion of cHtrA may be secreted out of the organisms but still trapped inside the inclusions. Both the intra-inclusion and cytosolic distribution of cHtrA were confirmed with a mAb against cHtrA (Figure 1B). Similar intra-inclusion stainings that are free of organisms were reported previously [15,57,58]. In contrast, most CPAF molecules were secreted out of the inclusions without obvious intra-inclusion accumulation. As expected, most of the chlamydial HSP60 molecules co-localized with the chlamydial organisms. The secretion of cHtrA into host cell cytosol became more obvious when the chlamydial inclusion membrane was counter-labeled using an anti-inclusion membrane protein antibody (Figure 1C). The cHtrA molecules were detected both inside and outside the inclusion membrane. The above observations together suggested that cHtrA might be secreted into both intra-inclusion space and the host cell cytosol.

**Figure 2:**
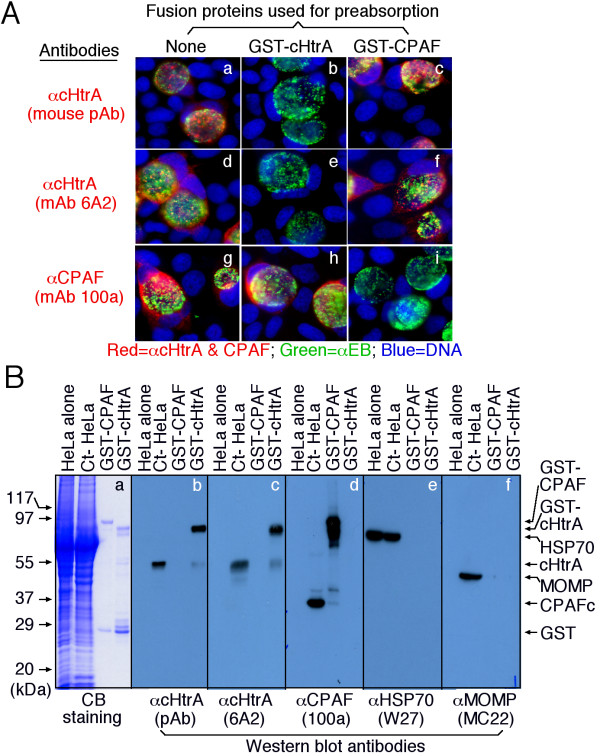
**The anti-GST-cHtrA fusion protein antibodies specifically detected the endogenous cHtrA produced by chlamydial organisms**. The anti-cHtrA antibodies with or without absorption with GST fusion proteins were used to detect the endogenous proteins *in C. trachomatis*-infected cells (A) and on nitrocellulose membranes (B). (A) *C. trachomatis*-infected cells were processed for immunostaining as described in Figure 1A legend. Note that the antibody labeling of endogenous antigens was blocked only by corresponding but not unrelated control fusion proteins. (B) In a Western blot assay, HeLa alone or HeLa infected with *C. trachomatis *(Ct-HeLa), GST-CPAF & GST-cHtrA fusion proteins were used as antigens as indicated on top of the figure. The antigens blotted onto nitrocellulose membrane were detected with mouse antibodies as displayed at the bottom of the figure. The anti-CPAF mAb 100a is specific to the C-terminal fragment of CPAF (CPAFc) and the full length CPAF is rapidly processed into the N- and C-terminal fragments to form intramolecular dimmers for activity during chlamydial infection. The control antibodies anti-MOMP and anti-human HSP70 were used to indicate that the Ct-HeLa samples contain chlamydial organisms and both HeLa and Ct-HeLa samples were loaded with similar amounts. Note that each antibody only detected a major protein band migrated at the molecular weight that matched the corresponding chlamydial or host proteins as indicated on the right side of the figure.

We next confirmed the antibody binding specificity by using an absorption procedure (Figure 2A). Both the intra-inclusion and host cell cytosolic signals detected by the anti-cHtrA antiserum or anti-cHtrA mAb 6A2 were removed by absorption with GST-cHtrA but not GST-CPAF fusion proteins. Similarly, the cytosolic signal detected with the anti-CPAF antibody was removed by absorption with the GST-CPAF but not GST-cHtrA fusion proteins, demonstrating that the anti-cHtrA and anti-CPAF antibodies specifically labeled the corresponding endogenous proteins without cross-reacting with each other. In a Western blot assay (Figure 2B), the anti-cHtrA antibodies recognized both the GST-cHtrA fusion protein and the endogenous cHtrA from the *C. trachomatis*-infected HeLa cells (Ct-HeLa) while the various control antibodies recognized the corresponding antigens without any significant cross-reactivity with each other. The anti-CPAF antibody detected the GST-CPAF fusion protein and also the C-terminal fragment (CPAFc) of the endogenous CPAF from the Ct-HeLa sample. CPAF is rapidly processed into the N- and C-terminal fragments during chlamydial infection and the mAb 100a is specific to the 35 kDa C-terminal fragment [26]. The anti-MOMP antibody detected MOMP from Ct-HeLa, confirming the presence of whole chlamydial organisms in the sample while the anti-human HSP70 antibody detected similar amounts of HSP70 in the HeLa alone and Ct-HeLa samples, indicating that an equivalent amount of whole cell lysates was loaded in both samples. These observations together have demonstrated that the anti-cHtrA antibodies only recognized cHtrA without cross-reacting with any other chlamydial or host cell proteins, suggesting that the cellular signals detected with the anti-HtrA fusion protein antibodies in the immunofluorescence assay were specific to the endogenous cHtrA produced by chlamydial organisms.

### 2. Secretion of cHtrA but not other chlamydial periplasmic proteins into host cell cytosol

**Figure 3:**
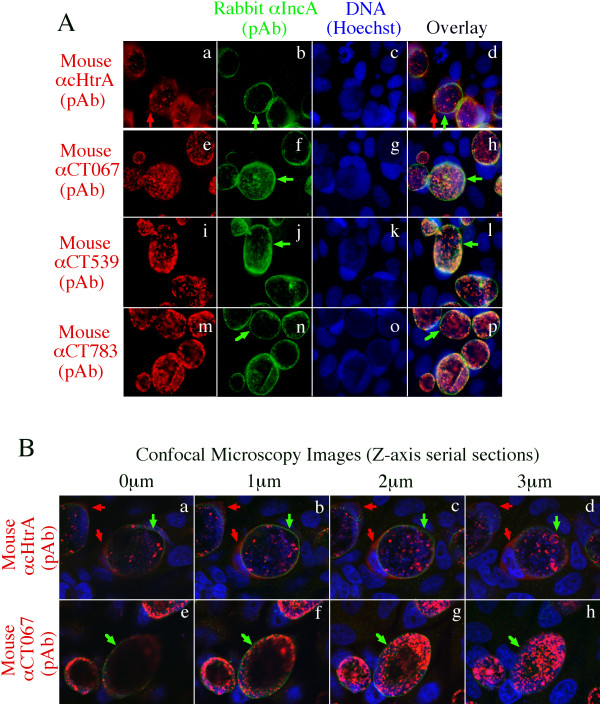
**The cHtrA but not other chlamydial periplasmic proteins are secreted into host cell cytosol**. HeLa cells infected with *C. trachomatis *organisms were processed and co-labeled with mouse antibodies against various periplasmic proteins (red) and a rabbit antibody against IncA (green) as described in Figure 1 legend. The Hoechst dye was used to visualize DNA (blue). The triple labeling was analyzed under a conventional fluorescence microscope (A) and confocal microscope (B). Under the confocal microscope, a series of four images were taken along the Z-axis by varying 1 μM between each. Note that cHtrA (red arrows) but none of the other periplasmic proteins including CT067, CT539 & CT783 was detected outside of the inclusion membrane (green arrows) by either immunofluorescence microscopy or confocal microscopy.

Since cHtrA is a periplasmic protein, we next tested whether localization in the host cell cytosol is a common characteristic of all chlamydial periplasmic proteins. The intracellular distributions of two periplasmic proteins involved in disulfide bond formation (CT539, TrxA or thioredoxin) and isomerization (CT783, PDI or protein disulfide bond isomerase; http://stdgen.northwestern.edu/) respectively and one periplasmic iron binding protein (CT067, YtgA, an ABC transporter system component; ref: [59,60]) were compared with that of cHtrA (Figure 3). Under a conventional fluorescence microscope (A), only cHtrA but not the other periplasmic proteins including CT067, CT539 & CT783 was detected outside of the chlamydial inclusions. This observation was confirmed under a confocal microscope (B). The Z-axis serial section images showed that cHtrA was clearly detected both inside and outside the inclusion membrane but CT067 was only detected inside the inclusion membrane.

**Figure 4:**
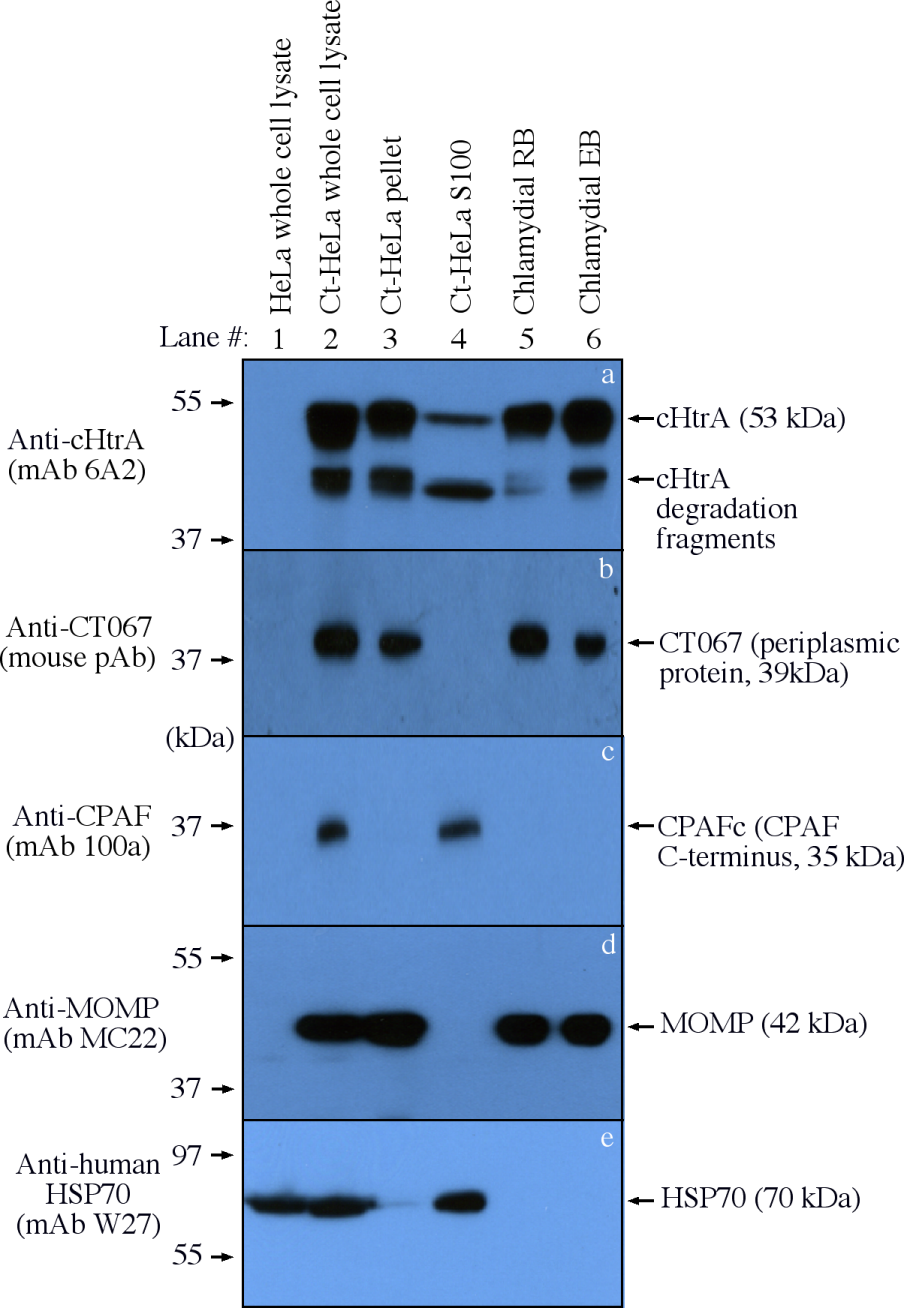
**The cHtrA but not CT067 is detected in the cytosolic fraction of the chlamydia-infected HeLa cells**. HeLa cells infected with *C. trachomatis *organisms (Ct-HeLa) were fractionated into nuclear (Ct-HeLa pellet, containing chlamydial inclusions, lane 3) and cytosolic (Ct-HeLa S100, containing chlamydia-secreted proteins, lane 4) fractions. The cellular fractions along with total cell lysates (normal HeLa, lane 1 & Ct-HeLa, lane 2) and purified chlamydial RB (lane 5) and EB (lane 6) organisms as listed at the top were resolved in SDS-polyacrylamide gels. The resolved protein bands were blotted onto nitrocellulose membrane for reacting with antibodies (listed on the left) against cHtrA (panel a), CT067 (b, a periplasmic iron binding protein), CPAF (c, a chlamydia-secreted protein), MOMP (d, a chlamydial outer membrane protein) and human HSP70 (e, a host cell cytosolic protein). All antibodies detected their corresponding proteins in the HeLa-L2 whole-cell lysate sample (lane 2) and other corresponding samples (as indicated on the right). Note that both cHtrA and CPAF but not CT067 or MOMP were detected in the cytosolic fraction (lane 4). CPAFc represents the C-terminal fragment of CPAF processed during chlamydial infection. The cHtrA degradation fragments (likely produced during in vitro sample processing) can always be detected with varying levels as HtrA is a powerful serine protease known to cleave itself [61] under certain conditions.

To directly visualize the molecular basis of the anti-cHtrA antibody-labeled cytosolic signals in *Chlamydia*-infected cells, the infected cells were fractionated into cytosolic (S100) and nuclear/inclusion (pellet) fractions. The distribution of cHtrA and CT067 in different fractions was compared in a Western blot (Figure 4). As a control for chlamydial proteins that are secreted into the host cell cytosol, CPAF was only detected in either the *Chlamydia*-infected whole cell lysate (Ct-HeLa) or cytosolic fraction (Ct-HeLa S100) samples but not other samples, which is consistent with what has been described previously [26]. Interestingly, cHtrA and its cleavage fragments but not CT067 was also detected in the cytosolic fraction, suggesting that cHtrA but not CT067 is secreted into host cell cytosol although both are periplasmic proteins. The cHtrA degradation fragments are likely generated during in vitro sample processing as HtrA is a powerful serine protease that is known to cleave itself [61]. To monitor the quality of the fractionation, the anti-MOMP antibody was used to indicate the pellet fraction that contains the chlamydial inclusions while an anti-human HSP70 antibody was used to indicate the host cell cytosolic fraction that contains the *Chlamydia*-secreted proteins. Detection with these antibodies revealed no cross contamination between the pellet and cytosolic fractions. In addition, detection with the anti-MOMP antibody also showed that the amounts of chlamydial organisms in the infected HeLa whole cell lysate, the pellet fraction and purified EB and RB samples were equivalent. These results together have independently confirmed that cHtrA is secreted into cytoplasm of *Chlamydia*-infected cells although it is also associated with the chlamydial RB and EB organisms.

### 3. Expression and secretion of cHtrA during chlamydial infection

**Figure 5:**
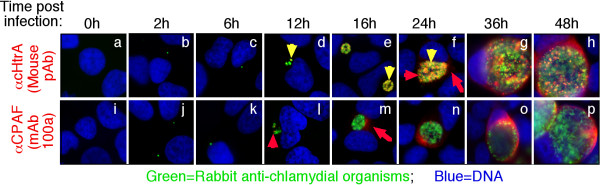
**Time course of cHtrA expression during *C. trachomatis *infection**. The *C. trachomatis*-infected culture samples were processed at various times after infection (as indicated on the top) for immunofluorescence staining as described in Figure 1 legend. The mouse anti-cHtrA (a to h) and anti-CPAF (mAb 100a; i to p) were visualized with a goat anti-mouse IgG conjugated with Cy3 (red) while the chlamydial organisms were visualized with a rabbit anti-chlamydia antibody plus a goat anti-rabbit IgG-Cy2 conjugate (green). Note that cHtrA was first detected inside the chlamydial inclusions at 12 hours after infection [panel d, yellow (overlapping with organisms) & red (free of chlamydial organisms) arrowheads], similar to the detection of CPAF. However, cHtrA secretion into host cell cytosol was only detected 24 h after infection while secretion of CPAF was already obvious by 16 h post infection.

**Figure 6:**
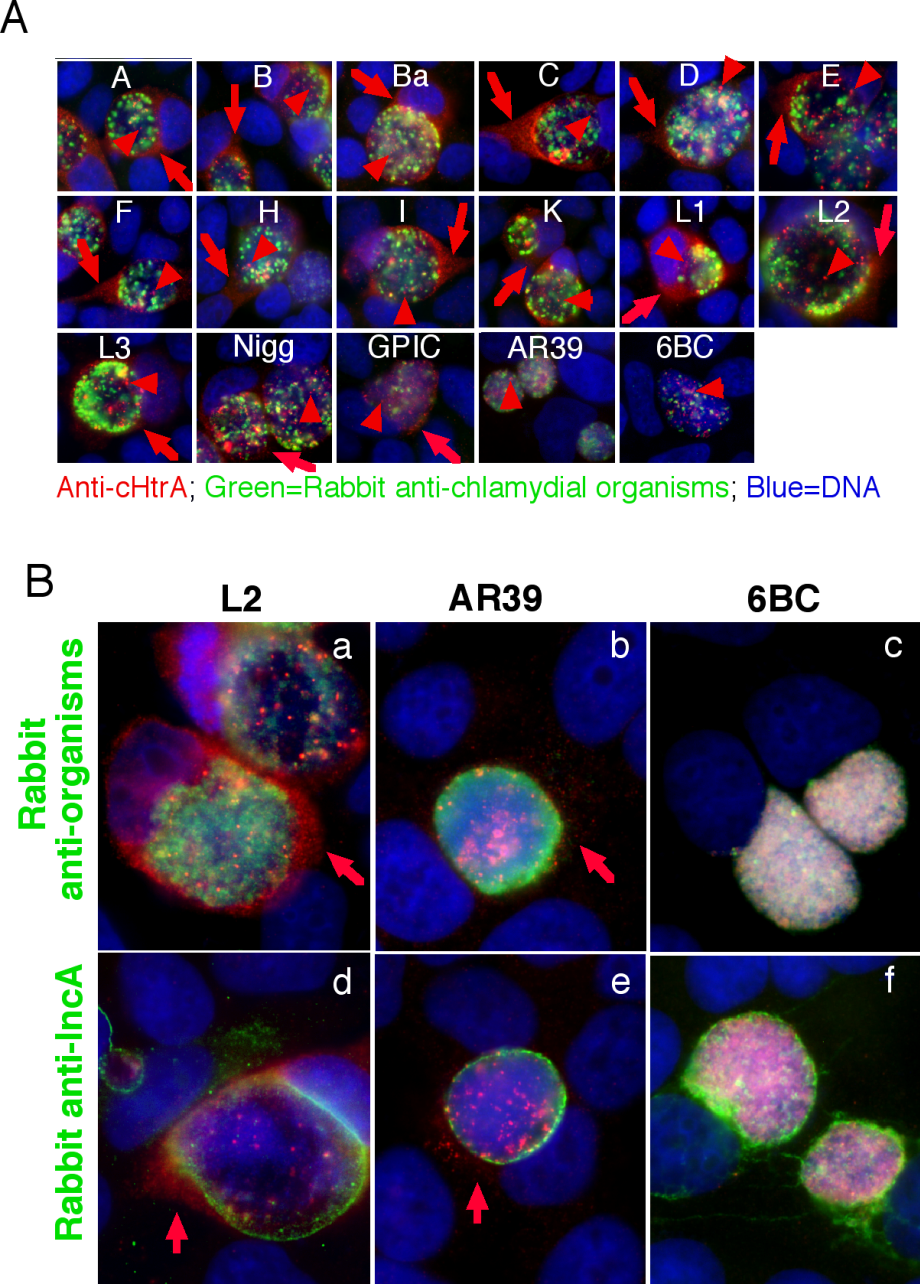
**Secretion of cHtrA into host cell cytosol by most chlamydial organisms tested**. HeLa cells infected with *C. trachomatis *serovars A, B, Ba, C, D, E, F, H, I, K, L1, L2, L3, *C. muridarum *Nigg strain, *C. caviae *GPIC, *C. penumonaie *AR39 isolate &*C. psiitaci *6BC organisms (as indicated in each panel) were processed at 40 h (all *C. trachomatis *serovars), 24 h (Nigg, GPIC & 6BC) or 72 h (AR39) after infection. (A) The processed samples were detected for cHtrA using the mouse anti-cHtrA fusion protein polyclonal antibody (red) in an immunofluorescence assay. The chlamydial organisms were visualized using a rabbit anti-CT395 fusion protein antibody (green) while the DNA was labeled with Hoechst dye (blue). Note that cHtrA was consistently detected in both the lumen of chlamydial inclusion (red arrowheads) and cytosol (red arrows) of cells infected with all *C. trachomatis *serovars and *C. muridarum *and *C. caviae *isolates. However, the cytosolic labeling of cHtrA was not clear in cells infected with *C. pneumoniae *AR39 and *C. psittaci *6BC organisms which were reexamined by co-staining with either anti-organisms (B, panels a-c) or anti-IncA (panels d-f) antibodies. Note that cytosolic cHtrA was detected in cells infected with *C. pneumoniae *AR39 (panels b & e) but not *C. psittaci *6BC organisms (c & f).

We further used the specific anti-cHtrA antibodies to characterize the endogenous cHtrA. As shown in Figure 5, cHtrA protein was detected inside the inclusions as early as 12 h after infection and secretion of cHtrA into host cell cytosol became apparent by 24 h post infection. Although CPAF was also detectable at 12 h, the secretion of CPAF was more robust and became very obvious as early as 16 h after infection. The cHtrA protein was detected both within the chlamydial inclusions and in the host cell cytosol while CPAF mainly accumulated in the host cell cytosol as infection progressed. Although both CPAF and cHtrA are serine proteases secreted by *C. trachomatis *organisms, their distinct secretion kinetics and intracellular distribution patterns suggest that they may fulfill different functions during chlamydial infection. To further evaluate whether cHtrA secretion is common to all chlamydial organisms, we monitored the cHtrA protein distribution in cells infected with various serovars and strains from different chlamydial species, including 13 *C. trachomatis *serovars and also isolates representing species of *C. muridarum*, *C. caviae, C. pneumoniae *and *C. psittaci *(Figure 6). The cHtrA protein was consistently detected in both the lumen of chlamydial inclusion and cytosol of host cells infected with all serovars of *C. trachomatis *organisms and isolates of *C. muridarum*, *C. caviae *and *C. pneumoniae *but not *C. psittaci*. Although secretion of cHtrA into the inclusion lumen and further into the cytosol of the infected cells seems to be a common feature of most chlamydial organisms tested, it is not known at this moment why the species *C. psittaci*, which primarily infect birds, failed to secrete cHtrA into host cytosol.

### 4. The secretion of chlamydial HtrA may require a type II but not type III secretion pathway

**Figure 7:**
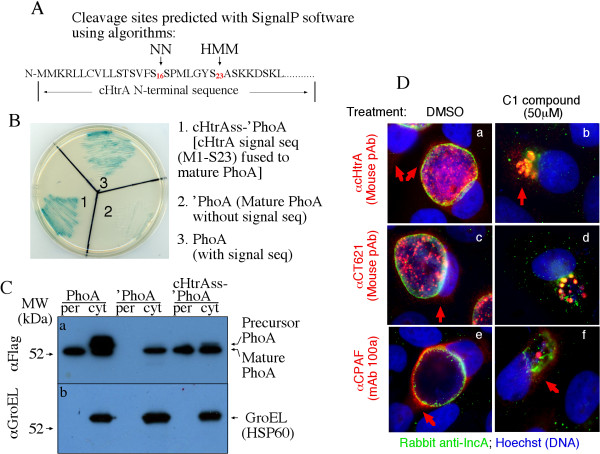
**cHtrA is secreted via a sec-dependent pathway**. **(A) **The SignalP 3.0 program with both the Neural Networks (NN) and Hidden Markov Model (HMM) algorithms http://www.expasy.ch was used to analyze the precursor cHtrA sequence from *C. trachomatis *serovar D http://stdgen.northwestern.edu/. The NN algorithm predicts a signal peptide from the first methionine residue (M1) to a serine residue at position 16 (S16) while the HMM-predicted signal peptide is M1-S23. **(B) **The M1-S23 peptide of cHtrA (cHtrAss) directed translocation of PhoA into bacterial periplasmic space (cHtrAss-'PhoA, slot 1, blue). Expression of the positive control full-length PhoA construct also led to the translocation of mature PhoA (with its intrinsic signal peptide, slot 3, blue) but the negative control mature PhoA construct failed to do so (without a signal peptide, 'PhoA, slot 2, white). (**C**) Bacterial transformants expressing the same three constructs were fractionated into periplasmic (per) and cytosolic (cyto) fractions and the fractions were detected with antibodies against a FLAG tag (anti-Flag, panel a) and GroEL (anti-GroEL, panel b) on a Western blot. Mature PhoA was secreted into the periplasm of bacteria expressing either the full-length PhoA construct or HtrAss-PhoA construct while mature PhoA stayed in the cytoplasm of the bacteria expressing the mature PhoA alone construct. (**D**) cHtrA secretion into the cytosol of chlamydia-infected cells is not inhibited by the type III secretion inhibitor C1 compound. HeLa monolayers infected with *C. trachomatis *L2 for 6 hr were treated with DMSO (panels a, c & e) or 50 μM C1 (b, d & f). Thirty-six hours after treatment, the cultures were processed for triply labeling with antibodies against IncA (green) and cHtrA, CT621 or CPAF (red) and DAPI for DNA (blue). C1 inhibited secretion of IncA and CT621 but not cHtrA or CPAF. Red arrows indicate chlamydia proteins that are secreted into host cell cytosol.

To determine the secretion pathway that chlamydial organisms may use to secrete cHtrA, we analyzed the amino acid sequence of cHtrA for secretion signal sequences using the program SignalP version 3.0 with NN (neural network) and HMM (hidden markov model) algorithms http://www.expasy.ch. Both NN and HMM algorithms predict an N-terminal signal peptide in cHtrA but with different cleavage sites. NN predicts a cleavage between S16 and S17 while HMM predicts the cleavage site between S23 and A24 (Figure 7A). We then tested the functionality of the cHtrA N-terminal sequence M1-S23 using a bacterium-based *phoA *gene fusion system (Figure 7B &7C). This assay system takes advantage of two characteristics of PhoA: the enzyme is only active after translocation into the bacterial periplasm, and the phosphatase activity can be conveniently monitored with the chromogenic substrate BCIP. DNA coding for the cHtrA N-terminal signal sequence covering residues M1 to S23 (designated as cHtrAss) was fused to the DNA sequence coding for mature PhoA (designated as 'PhoA). The fusion construct was expressed in pFLAG-CTC vector which adds a Flag epitope to the C-terminus of 'PhoA. The mature 'PhoA alone construct was used as a negative control while the precursor full-length PhoA (with its native N-terminal signal peptide) served as a positive control. As shown in Figure 7B, in the presence of BCIP, bacteria expressing either the precursor PhoA or the cHtrAss-'PhoA fusion constructs turned blue whereas bacteria expressing the mature PhoA alone ('PhoA) remained white, indicating that both the native PhoA and cHtrA signal peptides directed the translocation of PhoA into periplasm. We further used a Western blot analysis to monitor the distribution of PhoA protein in periplasmic (per) and cytosolic (cyto) fractions (Figure 7C). Mature PhoA was detected in the periplasm of bacteria expressing either the precursor PhoA or HtrAss-'PhoA fusion constructs while mature PhoA was only detected in the cytoplasm of the bacteria expressing the leaderless PhoA. Thus, the cHtrA N-terminal signal peptide is sufficient for directing PhoA across the bacterial inner membrane. We further found that the secretion of cHtrA was not inhibited by the C1 compound, an inhibitor known to inhibit chlamydial type III secretion system [52]. As positive controls, C1 inhibited the secretion of both IncA and CT621, two known chlamydial type III secretion substrates [30,52]. Consistently, the secretion of CPAF was not affected by C1. This is because secretion of CPAF is dependent on type II secretion pathway [62].

## Discussion

The obligate intracellular growth of *Chlamydia *requires the organisms to intimately interact with host cells. Secretion of chlamydial proteins into host cells is necessary for chlamydial organisms to ensure a safe intracellular niche for completing biosynthesis and producing progenies. Identifying chlamydial proteins that are secreted into host cell cytoplasm has been a productive approach for understanding chlamydial pathogenic mechanisms [20,22-31]. In the current study, we characterized the chlamydial serine protease cHtrA by localizing its intracellular distribution. We have presented convincing evidence that cHtrA is secreted out of the chlamydial organisms into both chlamydial inclusion lumen and cytosol of the infected cells. First, both the cHtrA fusion protein-specific polyclonal and monoclonal antibodies detected intracellular secretion patterns distinct from those of CPAF, another secreted serine protease by chlamydial organisms. The cytosolic signals were confirmed using inclusion membrane as a reference and under a confocal microscope. Second, the antibody labeling of cHtrA was removed by absorption with the cHtrA but not CPAF fusion proteins while the labeling of CPAF was removed by CPAF but not cHtrA fusion proteins, indicating that there was no cross-reactivity between anti-cHtrA and anti-CPAF antibodies. Third, in a Western blot with both HeLa alone and *Chlamydia*-infected whole cell lysates as antigens, the anti-cHtrA fusion protein antibodies detected a major protein band migrated at the molecular position expected for cHtrA, demonstrating that the anti-cHtrA antibodies specifically recognized the endogenous cHtrA without cross-reacting with any other cellular or chlamydial proteins. Fourth, the cytosolic cHtrA signals are likely due to active secretion but not passive leaking of cHtrA since various other abundant periplasmic proteins were not detected in the host cell cytosol. Finally, secretion of cHtrA into host cell cytosol was detected 24 h after infection while CPAF secretion occurred at 16 h after infection. Secretion of cHtrA was detected in most chlamydial species but not *C. psittaci*. These results together suggest that cHtrA secretion into host cell cytosol is a specific process and the secreted cHtrA may play an important role in chlamydial pathogenesis.

**Figure 8:**
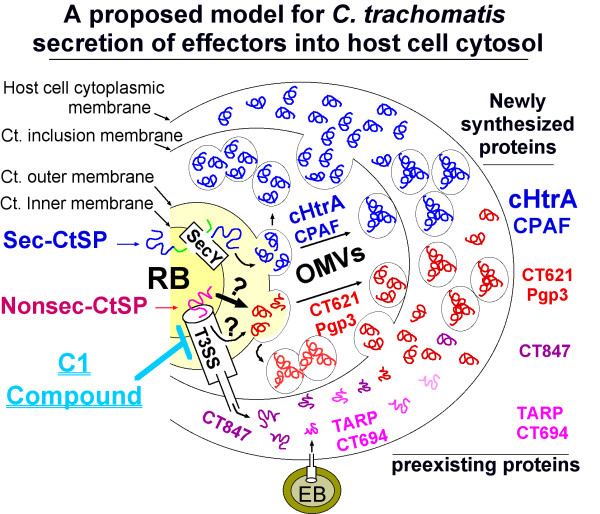
**A proposed model for *C. trachomatis *secretion of effectors into host cell cytosol**. When an infectious and metabolically inactive elementary body (EB) attaches to an epithelial cell, preexisting effectors such as TARP and CT694 can be injected into host cell cytosol via a single step type 3 secretion system (T3SS) for facilitating EB invasion. Once the internalized EB is differentiated into a non-infectious but metabolically active reticulate body (RB), newly synthesized chlamydial proteins can be secreted into host cell cytosol via either the single step T3SS (for example, secretion of CT847) or multi-step pathways. The *C. trachomatis*-secreted proteins (CtSPs) with an N-terminal signal sequence (termed Sec-CtSPs) such as cHtrA & CPAF may be translocated into periplasm via a SecY-dependent pathway while those without any N-terminal signal sequences (Nonsec-CtSPs) may be translocated into the periplasmic space via a novel translocon or a leaking T3SS pathway. The periplasmically localized CtSPs may exit the chlamydial organisms via an outer membrane vesicle (OMV) budding mechanism. The CtSP-laden vesicles in the inclusion lumen can enter host cell cytosol via vesicle fusion with or passing through the inclusion membrane. That's why CT621 can be visualized in granules in the lumen of inclusion and its secretion can also be inhibited by C1, a small molecule inhibitor known to target bacterial T3SS.

HtrA is a highly conserved serine protease present in the ER of eukaryotic and periplasmic space of bacterial cells. However, there has been no report on its secretion outside of eukaryotic or bacterial cells. Secretion of cHtrA out of chlamydial organisms may represent a unique feature *Chlamydia *has evolved during its interactions with host cells. A sec-dependent pathway may play an important role in exporting cHtrA into host cell cytosol since the N-terminal leader peptide of cHtrA is functional and the secretion is not inhibitable by a type III secretion inhibitor. However, The sec-dependent pathway can only translocate cHtrA into the periplasmic region. It is still unknown how the periplasmic cHtrA passes through the outer membrane to enter the chlamydial inclusion lumen and further into host cell cytosol. The same challenge also applies to the secretion of CPAF. A sec-dependent pathway is necessary for CPAF secretion [62]. Similarly, how the periplasmic CPAF crosses the outer membrane remains unclear. Since CPAF was detected in granules in the lumen of inclusions during the early stage of chlamydial intracellular growth, an outer membrane vesicular budding model has been proposed for CPAF secretion into host cell cytosol [62], which may also be suitable for the secretion of cHtrA (Figure 8). Evidence for supporting this hypothesis comes from the observation that cHtrA-laden granules/vesicles that are free of chlamydial organisms were readily detected in the chlamydial inclusions. Although it remains to be determined how exactly cHtrA or CPAF is secreted out of the organisms and into host cell cytosol, as more effector molecules are identified, more tools will be available for figuring out the secretion pathways *Chlamydia *has evolved for exporting virulence factors.

HtrA is a hexamer formed by two trimeric rings staggered on top of each other [46,47]. It possesses dual functions as both a chaperone and a protease [44]. Whether in eukaryotic ER or prokaryotic periplasmic space, HtrA can transmit the stress signals from unfold proteins into stress responses [48-51]. lt appears that *Chlamydia *can respond to various stress signals by regulating the expression levels of cHtrA [45]. Although it is still unknown how the periplasmic cHtrA works, these previous observations can at least suggest that cHtrA is functional during chlamydial infection. Nevertheless, a more important question relevant to the current study is what roles cHtrA has after it is secreted into host cell cytosol and whether the secreted cHtrA contributes to chlamydial pathogenesis. Can the secreted cHtrA gain access to host cell ER to regulate host unfolded protein stress responses? What cellular proteins the secreted cHtrA molecules target during chlamydial infection in the presence or absence of stress stimulation. Efforts are underway to address these questions.

## Conclusions

Secretion of chlamydial proteins into host cells is necessary for chlamydial organisms to establish and complete intracellular growth. Thus, identifying chlamydial proteins secreted into host cell cytoplasm has become a hot subject. Here, we have presented convincing evidence that the chlamydial periplasmic stress response serine protease cHtrA is secreted out of the chlamydial organisms into both chlamydial inclusion lumen and host cell cytosol. This secretion is specific since various other abundant chlamydial periplasmic proteins remained within the organisms. This novel finding suggests that the highly conserved cHtrA, in addition to its role in modifying chlamydial proteins in the periplasmic region, may also target host proteins, which is consistent with the overall concept that Chlamydia may use proteolysis as a powerful tool for manipulating host signaling pathways.

### Note added in proof

During revision of the manuscript, Hoy et al published a report on *Helicobacter pylori *HtrA as a new secreted virulence factor that cleaves E-cadherin to disrupt intercellular adhesion. Hoy et al. 2010. EMBO reports. 11:798-804.

## Authors' contributions

XW carried out most of the immunofluorescence and PhoA experiments; LL performed the confocal and Western blot assays as well as repeated some immunofluorescence assays; SG did the inhibitor experiment and carried out some immunofluorescence assays; RF participated in the immunofluorescence experiments; DC participated in the design of the experiments and also provided technical guidance to XW. GZ conceived of the study, and participated in its design and coordination and drafted the manuscript. All authors read and approved the final manuscript.
